# Evolving our understanding of cancer: an interview with Charles Swanton

**DOI:** 10.1242/dmm.052318

**Published:** 2025-03-12

**Authors:** Charles Swanton

**Affiliations:** The Francis Crick Institute, 1 Midland Road, London NW1 1AT, UK


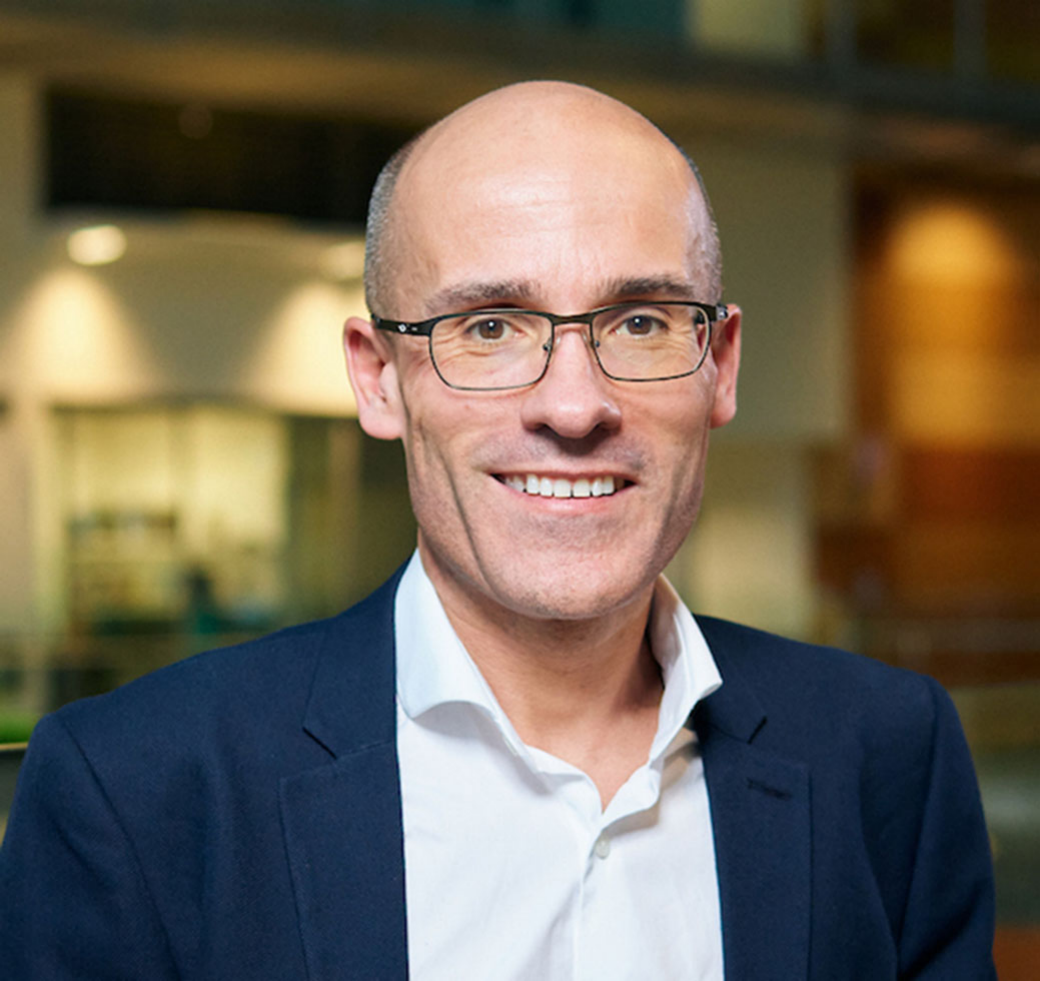


**Charles Swanton**

Professor Charles Swanton is a clinician scientist whose work is focused on understanding cancer evolution, with a particular focus on lung cancer. He is the Deputy Clinical Director at The Francis Crick Institute, UK, and Cancer Research UK (CRUK)’s Chief Clinician. His research has significantly advanced our knowledge of the cell-to-cell genetic variability within tumours and the molecular pathways that drive cancers to evolve, enabling them to spread and become resistant to therapy.

Charles completed his MD-PhD at the Imperial Cancer Research Fund Laboratories in 1998. After training as a CRUK-funded postdoctoral clinician scientist, he established his lab in 2008 at the London Research Institute, UK (now part of The Francis Crick Institute), alongside becoming a consultant oncologist at the Royal Marsden Hospital, UK. He was promoted to Chair in Personalised Cancer Medicine at University College London (UCL), UK; and Consultant Thoracic Medical Oncologist at UCL Hospitals in 2011. Charles is the Chief Investigator of the TRACERx clinical study which has been running since 2014 with the aim to track cancer evolution in a large cohort of patients. His work has also led to the creation of a spin-out company, Achilles Therapeutics, developing personalised T-cell therapies that target clonal mutations in lung cancer. In this interview, we discuss Charles' fascinating discoveries at the interface of clinical and laboratory research, the challenges of translating science to the clinic and the future of cancer prevention.

**What made you want to become a researcher as well as a clinician?**

When I was at medical school I did an undergraduate BSc in cell pathology, which combined immunology with cell biology. I found it utterly fascinating, quite honestly. I did a small lab project with Benny Chain on antigen presentation, which really got me hooked on lab work and exploring biological questions. Around the same time, I had a close family member suffer from an aggressive cancer that was ultimately cured thanks to a combination of chemotherapy and radiotherapy. I think the two together made me realise that research over decades preceding that diagnosis had helped cure him. As a result of that, I thought, “I want to be part of this”. I love the research and love the potential impact it could have on patients. I wanted to be involved in that discovery science endeavour *en route* to finding new therapies for patients suffering from cancer.

**How has your perspective as a clinician shaped your research?**

I think, as clinicians, we have a different but complementary view. I don't think it's exclusively required, but I think we have a slightly different overview of the clinical problems and how we can use discovery research in the laboratory to help address them. I often reflect on this actually: in the lab, the standard approach starts with a reductionist system and builds up our understanding. I'm working on a very complex system – human biology from patient data – and working my way down, so it's a top-down approach rather than a bottom-up. Both are important.

**Which key people in your field influenced you throughout your career?**

Lots, really! Where do I start? Bert Vogelstein – the field wouldn't be the same without him. He's made a tremendous impact on our understanding of cancer evolution, driver oncogenes, circulating tumour DNA, understanding cancer risk. Then, in the genome instability field, people like David Pelman have begun to unravel our understanding of how chromosomes segregate, what happens when this goes wrong, and how chromosomes can fracture at mitosis resulting in chromothripsis. This has enabled us to understand the patterns of genomic aberrations we're seeing in sequencing data. Contributions to our understanding of mutational signatures and somatic mutations in normal tissue from people like Mike Stratton, Peter Campbell and Inigo Martincorena – combined with Alan Balmain's work on the influences of chronic inflammation on tumour initiation and promotion – have changed the way I think about this disease in an immeasurable way.

There are also many clinical investigators who push the field forward in our understanding of combination chemotherapy, immunotherapy and targeted therapy, advancing cures for patients with cancer. That, combined with early detection, like Harry de Koning's work in low-dose computed tomography screening for patients at high risk of lung cancer, has shifted the disease from a late-stage diagnosis to an early-stage diagnosis that's resulting in more cures than ever before. So, it's a full spectrum from discovery science through to impact that I find fascinating.

I remember being blown away by those data and thinking how extraordinary it is that we have a kinase in our genome that's almost identical to one in yeast.

Undoubtedly, work from Paul Nurse provided staggering insights into the evolutionary conservation of cell cycle kinases and their impact on cell division and, ultimately, cancer. When I was a PhD student in the 1990s, I remember being blown away by those data and thinking how extraordinary it is that we have a kinase in our genome that's almost identical to one in yeast. It was hard to believe back then, and I still find it really quite something 30 years later. In many ways, that fostered and spurned my interest in evolution, both ecological evolution as well as cancer evolution. The pressure not to mutate the kinase in any way over hundreds of millions of years of evolution – it's just so fascinating that you end up with two kinases so identical in lower and higher-order eukaryotes, yeast and human. I think it's just mind boggling.

**You lead the TRACERx study which started in 2014 to track cancer evolution and monitor genetic variation within tumours in a large cohort of lung cancer patients. What have been the most important findings from studying this patient data over long time periods?**

It's been a really fascinating 10 years watching TRACERx evolve. It started from our observations in one or two patients that branched evolution appeared to be occurring in human tumours, and we now have data from TRACERx showing that branched evolution in cancer is ubiquitous: every tumour has one or more subclonal mutations that are present in only a subset of the tumour cells. I started off in this field 25 years ago where we were very familiar with more linear models of evolution, and now TRACERx and other studies have shown quite conclusively that tumours evolve in a branched manner. I guess it's obvious now, but 15 years ago, we had challenges at the review stage; it was not obvious to everybody. It was a theory put forward by Peter Nowell, but it hadn't been definitively proven at scale.

Another key finding is that there are subclonal driver events that lead to this branched evolution in many tumours, even untreated tumours. In other words, subclonal drivers don't just evolve because of treatment – they're there in nascent, treatment-naive tumours. This will likely impact the efficacy of targeted therapies.

Additionally, we could see very clearly that there is evidence of cancer immune predation throughout the evolution of the tumour, even at early stages. That was something Macfarlane Burnet and others had postulated but it hadn't been conclusively proven: that early-stage tumours would be subject to immune pressures. We can clearly see this in these tumours. For example, loss of human leukocyte antigens (HLAs) – cell surface markers important for immune function – occurs in one or more sub-clones in 40% of tumours in the TRACERx data. We've begun to explore this in much more detail, and we see evidence of transcription repression and alternative splicing of HLA, so we think these HLA molecules lose some of their antigen–T-cell receptor-binding abilities and become unstable or dysfunctional.

TRACERx has also unravelled the impact of chromosomal instability on patient outcome, showing that the more chromosomally unstable a tumour is, the worse the outcome. From evolutionary analysis, we've been able to pinpoint how chromosomal instability occurs in lung cancer. Whole-genome doubling events drive the diversification of cells at the ploidy level and help cells tolerate single chromosome mis-segregation events. We've also identified some of the driver genes implicated in that process. This includes loss of FAT atypical cadherin 1 (*FAT1*), which triggers whole-genome doubling and chromosome instability through homologous recombination deficiency.

We've also begun to better understand the ways in which somatic mutational diversity occurs through the induction of the cytidine deaminase apolipoprotein B mRNA editing catalytic polypeptide-like (APOBEC) that is activated later on in tumour evolution, we think by virtue of DNA replication stress. APOBEC drives these C to T mutations, which result in cell-to-cell variation, and we've shown in animal models that it drives more-rapid acquisition of drug resistance.

Most recently, we've got some very interesting insights into how all this starts in the beginning, and the role of tissue inflammation during lung cancer initiation through air pollution – i.e. how air pollutants get into macrophages and create chronic inflammatory stimuli through interleukin 1β, collaborating with progenitor cells that harbour oncogenic mutations to drive tumour initiation.

**Do you think air pollution could take over smoking as a primary driver of lung cancer in the future?**

The most important thing to say is that the risk of lung cancer from smoking is about 30-fold and the risk of lung cancer from air pollution is probably about 1.5- to 3-fold – depending on the concentration of ambient particulate matter – so the differences are huge. The biggest overwhelming risk factor for lung cancer today is smoking and, for the foreseeable future, it will continue to be. That said, air pollution is now considered to be the biggest cause of disability-adjusted life years globally – a measure of years lost to ill-health or premature mortality. Air pollution may contribute to 1000–3000 cases per year of lung cancer in the UK, whereas smoking will probably contribute to 30,000. It's a relatively small number compared to smoking, but the same can probably be said for cardiovascular disease and dementia etc. Across multiple disease pathologies together, it's a very large number of deaths resulting from air pollution. We're particularly concerned about air pollution because it's very hard to avoid. Humans have no choice over the air we breathe. There are parts of the world, particularly equatorial regions, that have much higher levels of air pollution. In part, this is due to heat and humidity as well as coal-fired power stations being localised close to the equator, so we are quite concerned about the impact on health.

**Your research has led to a spin-out company, Achilles Therapeutics, using T-cell therapy to target clonal mutations in lung cancer. How do you decide when a project or technology is mature enough for progression to a spin-out?**

That's a tough question. Thinking about the link between discovery and clinic, and just how proximal that link is to the clinic, is crucial. What steps would you need to go from this academic discovery to something that's of tangible value to a patient? That's not always easy, and that's why we need the help and support of our translation teams at the Crick, Cancer Research UK and UCL, as well as conversations with venture capital investors who will, ultimately, tell you whether or not your idea is too premature. Some of it is luck as well. Back in 2015, when we first started thinking about Achilles, there was a relatively large supply of money out there because interest rates were so low – things have become a lot tougher now. We were doing this at a very fortunate time in the money markets that meant there was financing available to get us started and to take our idea from discovery through to the clinic.

**What do you think the biggest barriers to translation are for scientists who would like to get their research to the clinic?**

The three main barriers are cost, time and practicality. It's very costly, whether getting a biomarker or a therapy into patients: processes like manufacturing and trials are very, very expensive. It also takes a long time to demonstrate that a new therapy, for example, is going to be of clinical value to a patient – this could be a 10-year process. By the time you've identified the target, discovered a lead molecule, gone through phase one, two and three trials, you're talking 10−15 years, possibly, and hundreds of millions of dollars of investment to get to that stage.

Scientists are very used to tinkering. We constantly tinker, constantly ask more questions, constantly optimise. […] when you go from the lab to the clinic through a translation opportunity, there is no more tinkering. You have to get to a clinical product as quickly as possible.

Another thing is that scientists are very used to tinkering. We constantly tinker, constantly ask more questions, constantly optimise. What I hadn't realised is when you go from the lab to the clinic through a translation opportunity, there is no more tinkering. You have to get to a clinical product as quickly as possible. And that's quite frustrating as a scientist because you constantly want to try and make things better, and it's not always possible. But you learn a lot. It's fascinating working with people from the commercial world. They teach you skills that you've not previously had. Coming up with a business plan, for example, I had no idea how to do that – deconstructing a scientific case and rebuilding it. The peer-review process for attracting money was harder than getting a paper published in a high impact journal. The quality of peer review that you get from investors who are investing their own money into a company is really extreme. It's like something I've never experienced before. So that was fascinating and stressful, but justifiably so. Patience is important because of the time it takes. You learn a lot about the commercial and the financial world in ways you wouldn't expect. It's a fascinating journey.

**What advancement do you think is going to have the biggest impact on cancer patients in the near future?**

Understanding how chronic inflammation triggers the initiation step of cancer formation from stem cells with activating oncogenic mutations has to be, in my view, the most important question. I am spending a lot of time trying to understand what these chronic inflammatory mediators are and how they act on the stem cell of origin, and how those cells that arise from the combined insults of the mutation and the chronic inflammatory stimulus might signal to other cells to, ultimately, form a cancer. What I think is happening is that this inflammation creates an epigenetic switch that irreversibly programs that stem cell to form a cancer stem cell. Opportunities might arise to prevent cancers from initiating in the first place if we understood which chronic inflammatory molecules to inhibit or how to reverse this process. I think this would open up a whole new field that I term ‘molecular cancer prevention’. In cardiology, for example, we use statins to prevent cardiovascular disease, but we don't really have any routine oral therapies that we can use to prevent cancer right now. The problem is that it's commercially tricky. Companies don't want to take on cancer prevention as a challenge because there's no commercial reimbursement model for it.

Ignore all advice because everybody is unique! […] do science because you're passionate about it and you love it.

**What is the best science-related advice you've received you would like to pass onto early-career researchers?**

Probably to ignore all advice because everybody is unique! No two careers are the same. I'd say, do science because you're passionate about it and you love it. If you don't love it and you don't find that you're blown away by discovering something that no one else has ever found before, then there are easier ways of making a living. I think you have to really enjoy it and really relish being in the lab working with colleagues to explore new avenues of research. That feeling when you're developing a gel, seeing a new blot or analysing some sequencing data and suddenly having a eureka moment, seeing something that nobody in the world has ever seen before – it's such an amazing feeling. It makes up for all the grant rejections, paper rejections and critical referees if you're a glass-half-full person.

**What do you like doing outside of your work?**

I do spend a lot of time thinking about work, and I do love work. When I'm not working, I'm either with my daughters, who are quite grown up now, with friends socialising or on my bike. I love cycling, I'm a big fan of road biking, both on my own and with friends. We've got a cycling group from work and we have a very good time going for rides together, it's a very social event.

